# Spina Bifida Occulta Is a Risk Factor for Spinal Cord Injury Without Fracture or Dislocation for Children Performing a Backbend During Dance

**DOI:** 10.3389/fped.2022.903507

**Published:** 2022-06-15

**Authors:** Guoqing Liu, Wei Jiang, Xiang Tang, Shali Tan, Mingqiang Zhang, Liang Tao, Nong Xiao, Yuxia Chen

**Affiliations:** ^1^Department of Rehabilitation, National Clinical Research Center for Child Health and Disorders, Ministry of Education Key Laboratory of Child Development and Disorders, Chongqing Key Laboratory of Pediatrics, Children's Hospital of Chongqing Medical University, Chongqing, China; ^2^Department of Pediatrics, The People's Hospital of Fengjie, Chongqing, China

**Keywords:** spinal cord injury without fracture or dislocation, spina bifida occulta, risk factor, children, China

## Abstract

**Objective:**

This study aimed to explore the clinical features and outcomes of children with spinal cord injury (SCI) without fracture or dislocation.

**Methods:**

The clinical data of children with SCI without fracture or dislocation in this retrospective study were collected in Chongqing, China (January 2010 to December 2021). We collected patient demographics at admission including age, gender, cause, level, and severity of the injury in admission and complications. Reports from radiologic imaging were reviewed to identify spina bifida occulta (SBO). Neurological function was evaluated using the American Spinal Injury Association (ASIA) Impairment Scale (AIS) for an SCI.

**Results:**

A total of 74 children with SCI (male, 27%; female, 73%; male-to-female ratio, 1:2.7; average age, 5.7 years) were included. The main cause of injury was backbend during the dance (34 patients, 45.9%, including 2 patients who hugged back falling backward), followed by traffic accidents (17 patients, 23%). Children with backbend-related SCI were older than other children (6.9 vs. 4.9 years old, *P* < 0.001). When reviewing all radiological images, it was found that 20 (27%) patients with SCI had SBO. The proportion of SCI with SBO caused by backbend was considerably higher than those caused by non-backbend (41.2 vs. 15%, *P* = 0.012). The AIS were 22 (29.7%), 4 (5.4%), 8 (10.8%), 31 (41.9%), and 9 (12.2%) in A, B, C, D, and E, respectively. The prognosis was poorer in the backbend during dancing than other causes of injury (*p* = 0.003).

**Conclusion:**

This study showed that backbend during the dance was the main cause of children's SCI without fracture or dislocation in Chongqing, China. The prognosis was poorer in those children than in other causes of injury. Meanwhile, we have established an association between SBO and SCI for children performing a backbend during the dance.

## Introduction

Spinal cord injury without radiologic abnormality (SCIWORA) was defined as traumatic myelopathy without radiographic features of spinal fracture or dislocation, which was initially described by Pang and Wilberger in 1982 (1). Along with the development and popularity of MRI, real SCIWORA or without neuroimaging abnormality has become increasingly rare. Previous studies demonstrated the leading etiologies of spinal cord injury (SCI) without fracture or dislocation, including motor vehicle accidents, falls, sports, and child abuse in western countries (2). While that appeared different in China, dance injury has been supposed to be the main cause (3, 4). In 2019, Liang et al. (5) reported three cases of pediatric SCI caused by minor trauma and suggest that spina bifida occulta (SBO) might be a predisposing factor for SCI without fracture or dislocation.

Spina bifida is the term used to describe the failure of fusion of the neural folds during the neurulation phase of embryologic development, which has usually been divided into spina bifida cystica (SBC) and SBO (6). SBO has recently been linked with voiding dysfunction, constipation, or lower urinary tract abnormality (7, 8). The pathological changes in the core of SBO are tethered spinal cord, which has longitudinal traction damage to the spinal cord (6). Chronic spinal cord traction may play an important role in the mechanism of pediatric SCI following minor trauma.

Therefore, the aim of the present study was to determine the demographic of SCI without fracture or dislocation and to compare the prevalence of SBO between SCI following backbend during the dance and those without backbend. Furthermore, the study also assessed potential risk factors for the severity of the injury. Identification of risk factors for children attending dancing courses could be of value in risk assessment and prevention.

## Materials and Methods

### Study Population

We retrospectively analyzed 74 children with SCI identified in the medical database at Children's Hospital of Chongqing Medical University from January 2010 to December 2021. Two independent researchers reviewed all medical records and radiological images to identify SCI. The inclusion criteria were as follows: (1) patients admitted with a diagnosis of traumatic spinal cord injury; (2) without fracture or dislocation of the spine; and (3) follow-up time >6 months. The exclusion criteria were as follows: (1) with any other neurologic diseases; (2) incomplete radiological data; and (3) with neoplasm, vascular malformation, and so on. The study was approved by the Ethics Committee of Children's Hospital of Chongqing Medical University (NO. 202249).

### Measures and Definition

We collected patient demographics at admission including age, gender, cause, level, and severity of the injury in admission, and complications. Reports from radiologic imaging were reviewed to identify SBO, if present, and the length of lesions on a sagittal T2-Weighted magnetic resonance image (MRI). Neurological function was evaluated using the American Spinal Injury Association (ASIA) Impairment Scale (AIS) for a spinal cord injury, the severity of the injury was graded as A to E, while A represents a complete injury, and B through E represents incomplete injuries. Age was divided into two groups: 0–8 years and 9–18 years. Falling below 1 meter was classified as a low fall, and above 1 meter was classified as a high fall.

### Statistical Analysis

Continuous variables were expressed as means ± *SD* and categorical variables as numbers and percentages. Differences in categorical variables were compared using Chi-square or Fisher's exact test between groups. The two means were compared with independent *t*-tests for normally distributed variables and the Mann-Whitney U test for nonnormally distributed variables. Univariate and multivariate logistic regression analyses were applied to assess the risk factors for the severity of the injury. A *P*-value of <0.05 was considered statistically significant. Statistical analyses were performed using R software version 4.1.2 (Project for Statistical Computing).

## Results

### Patients Characteristics

Patients characteristics are summarized in Table 1. For those subjects, the average age range was 5.8 (2.7) years old, and the maximal age range was 15 years, while the minimum age range was 1.1 years. The main etiology of injury was backbend during dancing (34 patients, 45.9%, including 2 patients hugged back falling backward), followed by traffic accident (17 patients, 23%). Only very few injuries (3 patients, 4.1%) occur during sports activities. In total, 65 (87.8%) patients had various lesions determined by MRI. The most common type of complication was the neurogenic bladder, which occurred in 45 of 54 patients (83.3%) during the convalescent phase. Urinary tract infection (66.7%) and osteopenia (48.1%) were the second and third most common complications found in patients, respectively.

**Table 1:** Clinical characteristics (*n* = 74).

Age (year), mean (SD)	5.8 (2.7)
Sex, *n* (%)	
Male	20 (27.0)
Female	54 (73.0)
Cause of injury, *n* (%)	
Back bend	34 (45.9)
Traffic accident	17 (23.0)
High fall	3 (4.1)
Low fall	15 (20.3)
Violence	2 (2.7)
Sports	3 (4.1)
MRI, *n* (%)	
Normal	9 (12.2)
Abnormal	65 (87.8)
Severity of the injury	
Complete	25 (33.8)
Incomplete	49 (66.2)
Complication in rehabilitation (*n* = 54), *n* (%)	
Neurogenic bladder	45 (83.3)
Osteopenia	26 (48.1)
Urinary Stone	6 (11.1)
Spasticity	14 (25.9)
Pressure ulcer/scald	7 (13.0)
Urinary tract infection	36 (66.7)
Progression at last follow up	
A	22 (29.7)
B	4 (5.4)
C	8 (10.8)
D	31 (41.9)
E	9 (12.2)

*SD, standard deviation*.

### Patients Characteristics: Backbend vs. Non-backbend

**Figure 1:**
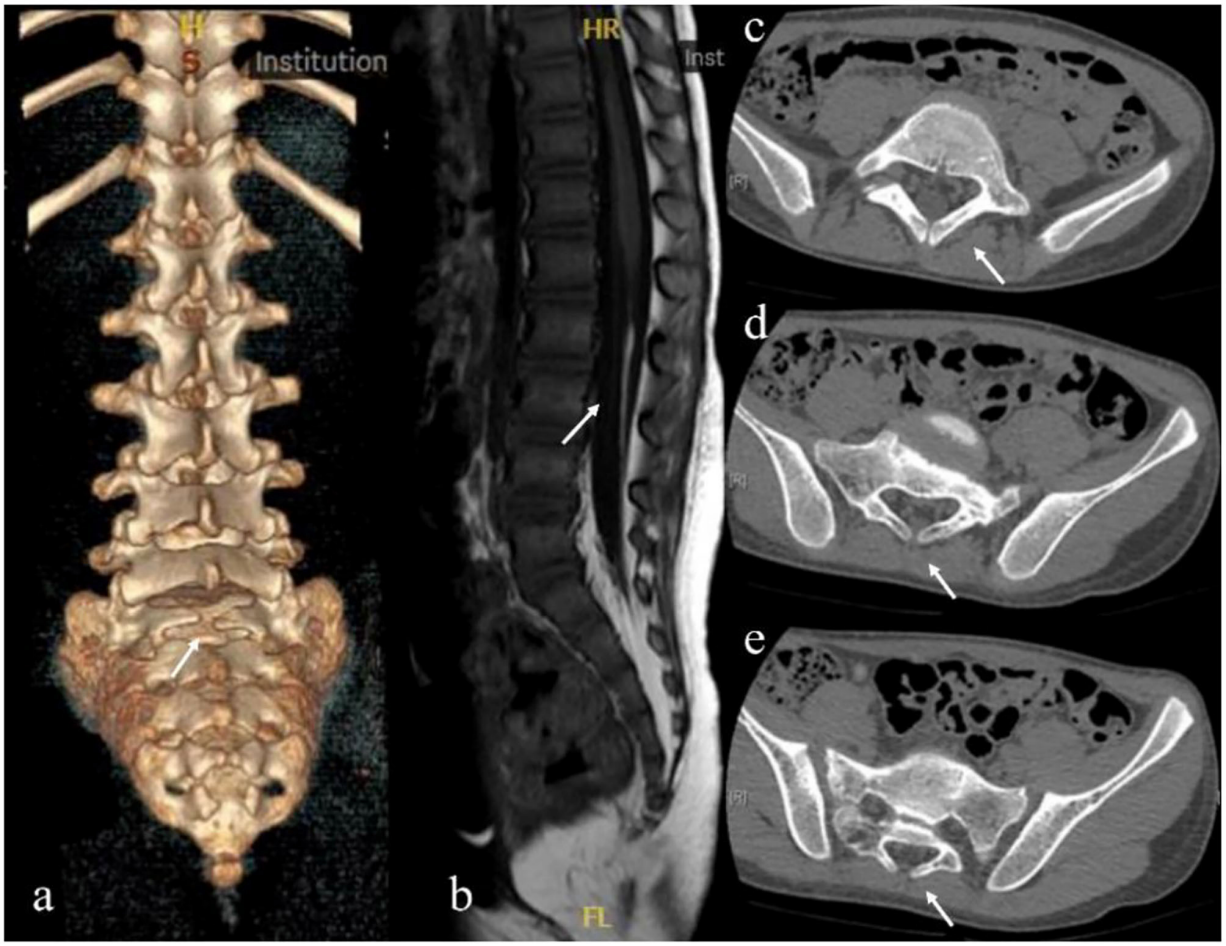
Radiological images showing spina bifida occulta. **(a)** Sacral 1 spina bifida occulta. **(b)** Fatty filum terminale. **(c–e)** are from the same patient. **(c)** Sacral lumbarization and Lumbar 6 spina bifida occulta. **(d)** Sacral 1 spina bifida occulta. **(e)** Sacral 2 spina bifida occulta.

Table 2 summarizes the demographic, injury, and MRI features of patients by backbend vs. non-backbend. The causes of injuries in patients by backbend were older than those caused by non-backbend (6.9 vs. 4.9 years old, *P* < 0.001). There was a proportional difference between males and females (female predominance), with a male-to-female ratio of 1:2.7. Injuries in patients caused by back bend were more likely to suffer from longer levels of lesions determined by MRI (7 vs. 5 vertebral). When reviewing all radiological images, we were surprised to find that there were 20 (27%) patients with SBO in all patients (Figure 1). The main types of SBO were simple lumbosacral spinal dysraphism (15 patients, 75%, including one patient who had combined sacral lumbarization) and fatty filum terminale (4 patients, 20%). The proportion of patients with SBO caused by backbend was considerably higher than those caused by non-backbend (41.2 vs. 15%, *P* = 0.012).

**Table 2:** Characteristics of SCI caused by backbend and non-backbend.

	Backbend *n* = 34	Non-backbend *n* = 40	*P*
Age, mean (SD)	6.9 (1.6)	4.9 (3.1)	<0.001
Sex			<0.001
Male	1 (2.9)	19 (47.5)	
Female	33 (97.1)	21 (52.5)	
Neurological level of injury			<0.001
Paraplegia	34 (100)	28 (70.0)	
Tetraplegia	0	12 (30.0)	
Severity of the injury			0.455
Complete	13 (38.2)	12 (30.0)	
Incomplete	21 (61.8)	28 (70.0)	
Length of lesion, Median (IQR)	7 (3, 9)	5 (3, 7.5)	0.268
SBO			0.012
Yes	14 (41.2)	6 (15.0)	
No	20 (58.8)	34 (85.0)	

*SD, standard deviation; IQR, interquartile range; SBO, spina bifida occulta*.

### Risk Factors for Severity of the Injury

Univariate and multivariate logistic regression analyses were employed to analyze the risk factors that featured statistical differences between the complete SCI group and the incomplete SCI group. It was observed that sex, age, cause, and SBO are not the risk factors affecting the severity of the injury, as shown in Table 3.

**Table 3:** Univariate and multivariate logistic regression analysis of the severity of the injury.

**Variables**	**Univariate**			**Multivariate**		
	**OR**	**95%CI**	***P***	**OR**	**95%CI**	***P***
Sex (female/male)	0.689	0.238–1.995	0.493	0.443	0.119–1.651	0.225
Age (>8/ ≤ 8)	0.847	0.233–3.078	0.800	0.864	0.233–3.204	0.826
Cause (backbend/non-backbend)	1.444	0.549–3.800	0.456	2.368	0.690–8.131	0.171
SBO (yes/no)	0.789	0.261–2.390	0.676	0.682	0.211–1.201	0.522

*OR, odds ratio; CI, confidence interval; SBO, spina bifida occulta*.

### Progression During the Last Follow-up

American Spinal Injury Association Impairment Scale grades and complications at the last follow-up were shown in Table 4. Final AIS grades were as follows: 22 A (29.7%), 4 B (5.4%), 8 C (10.8%), 31 D (41.9%), and 9 E (12.2%). The prognosis was poorer in the backbend during dancing than other causes of injury (*p* = 0.003). Complete injury in admission was also significantly associated with poorer neurologic recovery (*p* < 0.001). Although there was no statistical difference between SBO and normal, the proportion of patients with SBO caused by backbend was considerably higher than that caused by non-backbend (41.2 vs. 15%, *P* = 0.012).

**Table 4:** Progression at last follow-up.

	**AIS**						**P**
	**ALL (*n* = 74)**	**A (*n* = 22)**	**B (*n* = 4)**	**C (*n* = 8)**	**D (*n* = 31)**	**E (*n* = 9)**	
Age							0.880
0–8 year	61 (82.4)	18 (81.8)	4 (100)	6 (75.0)	26 (83.9)	7 (77.8)	
9–18 year	13 (17.6)	4 (18.2)	0	2 (25.0)	5 (16.1)	2 (22.2)	
Sex							0.069
Male	20 (27.0)	3 (13.6)	3 (75.0)	3 (37.5)	10 (32.3)	1 (11.1)	
Female	54 (73.0)	19 (86.4)	1 (25.0)	5 (62.5)	21 (67.7)	8 (88.8)	
Etiology							0.003
Backbend	34 (45.9)	14 (63.6)	0	1 (12.5)	12 (38.7)	7 (77.8)	
Non-back bend	40 (54.1)	8 (36.4)	4 (100)	7 (87.5)	19 (61.3)	2 (22.2)	
Neurological level of injury							0.232
Paraplegia	62 (83.8)	21 (95.5)	4 (100)	7 (87.5)	23 (74.2)	7 (77.8)	
Tetraplegia	12 (16.2)	1 (4.5)	0	1 (12.5)	8 (25.8)	2 (22.2)	
Severity of the injury							<0.001
Complete	25 (33.8)	19 (86.4)	4 (100)	1 (12.5)	1 (3.2)	0	
Incomplete	49 (66.2)	3 (13.6)	0	7 (87.5)	30 (96.8)	9 (100)	
SBO							0.531
Yes	20 (27.0)	6 (27.3)	0	3 (37.5)	7 (22.6)	4 (44.4)	
No	54 (73.0)	16 (72.7)	4 (100)	5 (62.5)	24 (77.4)	5 (55.6)	

*SD, standard deviation; IQR, interquartile range; SBO, spina bifida occulta*.

## Discussion

There is a wide disparity in the incidence rates of SCI without fracture or dislocation across the world (9, 10), with the highest incidence in pediatrics subjects, which is largely explained by differences in spinal biomechanical specificities. Pediatrics show a great deformation capacity due to their disc and ligament elasticity and anatomic features, especially before 8 years of age (11, 12). Although some studies have investigated the pathogenesis of SCI caused by backbend during dance (13, 14), the underlying mechanisms remain incompletely understood.

Compared with previous studies in other countries, the obvious difference was the demographic factors, which were in accordance with the results of Zou et al. (4). The age of SCI for this study was younger than compared with other studies (5.8 vs. 9.78 years), besides, the male-to-female ratio was 1:2.7, indicating a slightly higher incidence of females compared with other countries in which the male-to-female ratio was 1.8–3.3:1 (2, 9). Alternatively, the incidence of paraplegia caused by thoracic or/and lumbar was higher (83.8%), this result reached 100% in patients with injuries caused by backbend during the dance, which was different from almost all of the previous studies as well (2, 9, 15). Road accidents (37%) and sports accidents (31%) were identified as the main causes of SCI by a systematic review (16). While backbend during the dance was the main cause of children SCI in China in the present study which is similar to the study by Zou et al. (4).

Previous literature suggested that children (particularly those younger than 8 years) have specific biomechanics of the vertebral column due to lack of uncus, anterior vertebral wall immaturity, and vertebral ligament elasticity (17). Under external force, vertebral displacement recaptures quickly, and damage to the spinal cord could occur at the same time. The present study identified that children younger than 8 years of age represented 82.4% of all children with SCI. Interestingly, demographic differences mentioned above were associated with the main cause. Attending dancing courses is a more common phenomenon for girls, especially at the age of 4–8 years. The spinal cord has poor tolerance to traction compared with the spinal. When the amplitude of back extension continues to rise beyond the tolerance threshold of the spinal cord which is fixed in vertebral foramen by spinal nerve root passing through the corresponding intervertebral foramina, a spinal cord injury of the thoracic and/or lumbar will be triggered (13). However, we did not find that backbend during the dance was an independent risk factor for SCI without fracture or dislocation. This may be due to the small sample size. In the current study, there was a significant difference in the prognosis between the backbend and non-backbend (*P* = 0.003). This was potentially due to more patients with complete injuries which also served as a poor prognostic indicator of SCI in the group backbend.

Another important finding was that 20 (27%) patients with SBO observed in our study included simple lumbo-sacral spinal dysraphism, fatty filum terminale, and sacral lumbarization for the first time. Spina bifida is the term used to describe the failure of fusion of the neural folds during the neurulation phase of embryologic development, which is usually been divided into SBC and SBO (18). Previous literature has commonly believed that SBO is a failure of fusion of the vertebral arches that are covered by the skin and mostly does not involve the spinal cord. Based on their pathological characteristics, SBO is classified into different types, including simple lumbosacral spinal dysraphism, spinal cord lipoma, diastematomyelia, fatty filum terminale, intradural lipoma, and so on (19). The pathological changes in the core of spina bifida are tethered spinal cord, that has longitudinal traction damage to the spinal cord (18). Lipomas of the spinal cord and fatty myelomeningocele often have fat masses that compress the spinal cord laterally and may restrict the growth and extension of spinal cord nerve roots, leading to neurodevelopmental dysplasia (19). SBO is easily disregarded since it does not show any symptoms, and even in those who show symptoms, the symptoms tend to be limited to only bowel and bladder dysfunction. Meanwhile, the proportion of SCIWORA with SBO caused by backbend was considerably higher than that caused by non-backbend (*P* = 0.012). We hypothesized that there was a possible association between SBO and spinal cord abnormalities which could cause spinal cord damage more easily. The hypothesis is supported by Liang et al. (5), who reported three children with real SCIWORA caused by minor trauma. Although three patients had no signs of tethered cord syndrome prior, tight filum terminale was found during the operation. The filum terminale is a fibrous and viscoelastic band that connects the conus medullaris to the periosteum of the coccyx, and it is thought to fixate and stabilize the lower cord during abnormal cephalad and caudal traction, and also allow the conus to move during flexion and extension of the spine (20). The fatty filum terminale refers to a filum that is both >2 mm in diameter and contains a fat signal (21). The fat infiltration can cause stretching and tethering of the conus resulting in neurological symptoms. Identification of risk factors for children attending dancing courses could be of value in risk assessment and prevention. Although the direct evidence is still missing and the exact mechanism remains unclear, SBO might cause chronic spinal cord traction or underlying structures occurs resulting in a pediatric thoracic and lumber SCI following minor trauma.

This study still has some limitations. First, the study was retrospective, thus, our results need confirmation in a prospective study. Second, single-center research study with inevitable selection bias. Third, we were unable to assess the risk factors for backbend during dance and SCI without fracture or dislocation due to the lack of a control group. Although we looked for risk factors for complete SCI, it was observed that sex, age, cause, and SBO are not the risk factors affecting the severity of the injury. This may be due to the small sample size. Further studies with larger sample sizes are required for confirming the relationship. Finally, lack of pathologically confirmed patients. However, all of the patients were strictly screened with both comprehensive ASIA assessments and radiographic. SCI without fracture or dislocation is a disease that has low prevalence rates but significant disability for children, and its poor prognosis severely influences the affected patients. It is important to prevent this disease. Therefore, scientific popularization should be strengthened to enhance the awareness of individuals who practice any activity that involves backbend. Children, particularly those younger than 8 years, should be encouraged to avoid prolonged, repetitive backbend of the spine to reduce the risk during the dance.

In summary, this study showed that backbend during the dance was the main cause of children's SCI without fracture or dislocation in China. The prognosis was poorer in those children than in other causes of injury. Meanwhile, we have established an association between SBO and SCI for children performing a backbend during the dance.

## Data Availability Statement

The raw data supporting the conclusions of this article will be made available by the authors, without undue reservation.

## Ethics Statement

The studies involving human participants were reviewed and approved by the Ethics Committee of Children's Hospital of Chongqing Medical University. Written informed consent from the participants' legal guardian/next of kin was not required to participate in this study in accordance with the national legislation and the institutional requirements.

## Author Contributions

GL conceptualized and designed the study, carried out the initial analyses, and drafted the initial manuscript. ST, MZ, and LT designed the data collection instruments and collected data. XT coordinated and supervised data collection, assisted in the statistical analysis, and carried out the initial analyses. WJ coordinated and supervised data collection and critically reviewed the manuscript for important intellectual content. YC and NX conceptualized and designed the study, supervised data collection, and reviewed and revised the manuscript. All authors read and approved the final manuscript.

## Funding

This work was supported by the Program for Technological Innovation and Application Development of Chongqing (cstc2019jscx-msxmX0197).

## Conflict of Interest

The authors declare that the research was conducted in the absence of any commercial or financial relationships that could be construed as a potential conflict of interest.

## Publisher's Note

All claims expressed in this article are solely those of the authors and do not necessarily represent those of their affiliated organizations, or those of the publisher, the editors and the reviewers. Any product that may be evaluated in this article, or claim that may be made by its manufacturer, is not guaranteed or endorsed by the publisher.
